# Aducanumab produced a clinically meaningful benefit in association with amyloid lowering

**DOI:** 10.1186/s13195-021-00838-z

**Published:** 2021-05-10

**Authors:** Jeffrey Cummings, Paul Aisen, Cynthia Lemere, Alireza Atri, Marwan Sabbagh, Stephen Salloway

**Affiliations:** 1 Chambers-Grundy Center for Transformative Neuroscience, Department of Brain Health, School of Integrated Health Sciences, University of Nevada Las Vegas (UNLV), Las Vegas, NV USA; 2 University of Southern California, San Diego, CA USA; 3 Ann Romney Center for Neurologic Diseases, Brigham and Women’s Hospital, Harvard Medical School, Boston, MA USA; 4 Banner Sun Health Research Institute, Banner Health, Sun City, AZ USA; 5 Center for Brain/Mind Medicine, Department of Neurology, Brigham and Women’s Hospital, Harvard Medical School, Boston, MA USA; 6 Cleveland Clinic Lou Ruvo Center for Brain Health, Las Vegas, NV USA; 7 Butler Hospital and Brown University, Providence, RI USA

**Keywords:** Aducanumab, FDA, Clinical trials, Monoclonal antibodies, Lecanemab, Donanemab, Gantenerumab

Aducanumab is a monoclonal antibody targeting amyloid beta protein (Aß), a defining feature of the biology of Alzheimer’s disease (AD) [1]. Laboratory studies showed high affinity of aducanumab for the neurotoxic oligomeric species of Aß [2]. Following a promising phase 1B trial [3], the sponsor (Biogen) implemented two phase 3 studies—EMERGE and ENGAGE. A planned futility analysis concluded that the treatment was not beneficial, and the trials were terminated. With the accrual of additional blinded data, the prespecified analysis of the primary outcome—Clinical Dementia Rating Sum of Boxes (CDR-sb)—showed that the EMERGE trial met its primary outcome and the ENGAGE trial did not. Biogen submitted the data to the US Food and Drug Administration (FDA) for review and possible marketing approval, setting the stage for a vigorous dialogue on aducanumab [4, 5].

The CDR-sb, comprising the primary outcome of ENGAGE and EMERGE, is a composite measure with cognitive and functional components including home activities, problem solving, and community engagement—skills highly valued by patients [6]. In EMERGE, aducanumab treatment resulted in a significant 22% slowing of decline on the CDR-sb [7]. This instrument has a restricted range (0–18); small changes reflect meaningful clinical alterations. In both trials, participants who received at least 14 doses of the highest dose of aducanumab showed similar levels of slowing on the CDR-sb (30% in EMERGE, 27% in ENGAGE). In EMERGE, all secondary measures including the Mini Mental State Examination, Alzheimer’s Disease Assessment Scale-cognitive subscale, and the Alzheimer’s Disease Cooperative Study Activities of Daily Living (ADCS ADL) scale showed statistically significant drug-placebo differences. The ADCS-ADL scale showed a robust 40% slowing of functional decline in the treatment group compared to the placebo group [7]. The Neuropsychiatric Inventory (NPI) that assesses an array of behavioral changes common in AD showed an 87% reduction from baseline scores in the high dose group of EMERGE [8]. There was a corresponding 84% reduction in caregiver distress. Disease-modifying therapies change the trajectory of disease progression; benefits observed in trials are anticipated to increase with long-term treatment. Extending the mild cognitive impairment stage of AD and delaying the dementia stage is very meaningful for a 68-year-old grandmother seeking to preserve daily activities, hobbies, and community and family engagement.

Amyloid plaques measured by amyloid positron emission tomography (PET) were markedly decreased by aducanumab in both trials. Phosphorylated tau (p-tau) in the cerebrospinal fluid (CSF) and medial temporal neurofibrillary tangles measured by tau PET in a small subset of patients were reduced as predicted by “the amyloid hypothesis.” Phosphorylated tau is closely linked to cognitive decline [1]. Statistically significant correlations were present between Aß reduction and the clinical outcomes of EMERGE and between Aß reduction and CSF p-tau changes [8].

An argument marshaled against accepting the EMERGE trial as evidence of efficacy is that previous clinical trials of drugs targeting Aß have been negative [4]. This view fails to account for recent promising clinical trials specifically involving anti-Aß monoclonal antibodies including lecanemab [9], gantenerumab [10], and donanemab [11] and the many learnings that have occurred concerning dose, targeting specific types of Aß, and treating patients earlier in the disease [12]. Doses of monoclonal antibodies have more than quadrupled from those used in previous trials as more evidence has informed exposure requirements, and study populations have shifted toward earlier intervention prior to extensive irreversible neurodegeneration. The recently reported positive donanemab phase 2 trial, linking reduction of brain amyloid with cognitive/functional benefit [11], provides particularly strong support for the therapeutic approach of aducanumab. In view of these recent findings, EMERGE results can be considered consistent with other similar studies rather than as an anomaly.

Aducanumab and several other monoclonal antibodies are associated with amyloid-related imaging abnormalities (ARIA) thought to represent effusion through the blood-brain barrier (ARIA-E) or hemorrhages (ARIA-H) associated with blood-brain barrier compromise. ARIA-E occurred in 34% and 35.5% of those receiving high-dose aducanumab in EMERGE and ENGAGE respectively. Most (80%) ARIA events are without symptoms. When symptoms occur, they include headache, dizziness, visual disturbances, and nausea. ARIA is a manageable side effect of treatment with aducanumab and far less compromising than complications of many routinely used cancer therapies.

Although not directly relevant to determining the efficacy and safety of aducanumab, criticism has been directed at the FDA for working too closely with Biogen in the submission process [13]. FDA has provided written guidance for regularly scheduled meetings with all sponsors and works closely with sponsors to ensure clear communication regarding trial expectations and outcome interpretations [14]. The FDA decision regarding aducanumab carries great significance for patients with AD and their families; close communication is required to reassure those whose lives could be altered by such a therapy that all due considerations have been observed.

In the EMERGE trial, aducanumab met its primary outcome and had beneficial effects on cognition, function, and behavior. Benefits were observed in ENGAGE participants who were treated with the high dose for longer periods. A decision not to approve aducanumab in spite of these outcomes will adversely affect the field of AD treatment research, discouraging biopharmaceutical companies from investing in this area [15]. The first treatment of AD, tacrine, had flaws, but it was a breakthrough that demonstrated the possibility of improving cognition in AD and was soon followed by improved and now widely used agents. We anticipate a similar reinvigoration of AD treatment research if aducanumab becomes publicly available.

An FDA Advisory Committee voted to recommend that the Agency not approve aducanumab based on a single positive study [4]. We believe that the perspective of the panel was too narrow, ignoring important scientific and clinically meaningful considerations. Based on the review of the totality of the data and our extensive experience with AD trials, research, and clinical care of patients and families, we conclude that aducanumab achieves the standard of meaningful efficacy with adequate safety in early AD. We support providing persons with AD, who face a progressive and incurable disease, with the option of making informed choices about their health and lives with respect to a first-generation drug with aducanumab’s risk-burden/benefit profile.

## Data Availability

Not applicable.

## Abbreviations

Aβ: Amyloid beta protein
AD: Alzheimer’s disease
ADCS-ADL: Alzheimer’s Disease Cooperative Study Activities of Daily Living
ARIA: Amyloid-related imaging abnormalities
ARIA-E: Amyloid-related imaging abnormalities—effusion
ARIA-H: Amyloid-related imaging abnormalities—hemorrhages
CDR-sb: Clinical Dementia Rating Sum of Boxes
CSF: Cerebrospinal fluid
FDA: US Food and Drug Administration
NPI: Neuropsychiatric Inventory
PET: Positron emission tomography
p-tau: Phosphorylated tau
